# Rotavirus-associated acute diarrhea outbreak in West Shewa Zone of Oromia Regional State, Ethiopia, 2017

**DOI:** 10.11604/pamj.2019.32.202.18188

**Published:** 2019-04-26

**Authors:** Abyot Bekele Woyessa, Almaz Abebe, Berhane Beyene, Mesfin Tefera, Esete Assefa, Hiwot Ketema, Birke Teshome, Ayenachew Bekele, Yohanis Dugasa, Shambel Habebe, Zewdu Assefa, Diriba Sufa, Dagnachew Alemu, Habtamu Tilahun, Mengistu Biru, Gemechu Shume

**Affiliations:** 1 Center for Public Health Emergency Management, Ethiopian Public Health Institute, Addis Ababa, Ethiopia; 2 Oromia Regional Health Bureau, Addis Ababa, Ethiopia

**Keywords:** Rotavirus, diarrhea, outbreak, genotype, vaccine, Ethiopia

## Abstract

**Introduction:**

Rotavirus causes severe-diarrheal diseases in infants. An estimation of 138 million rotavirus-associated diarrheal cases and 215,000 deaths occur every year globally. In December 2016, West-Shewa zone in Ethiopia reported unidentified gastrointestinal diarrhea outbreak. We investigated to identify the causative agent of the outbreak to support response operations.

**Methods:**

Medical records were reviewed, and the daily line list was collected from health facilities. Descriptive data analysis was done by time, person and place. Stool specimens were first tested by antigen capture enzyme immunoassay (EIA) technique and further confirmed by reverse-transcription polymerase chain reaction (RT-PCR) as a gold standard. The product of RT-PCR was genotyped for each gene using G1-G4, G8-G9 and G12 primers for VP7 gene and P(4), P(6), P(8) and P(14) primers for VP4 gene.

**Results:**

A total of 1,987 diarrheal cases (5.7 per 1000) and five deaths (case-fatality rate 0.25%) were identified and epidemiologically-linked to confirmed rotavirus from December 2016 to February 2017. Among the cases, 1,946 (98%) were < 5 children. Fourteen (74%) of the 19 tested stool specimens were positive for rotavirus by EIA and RT-PCR. Majority of strains detected were G12P(6) (25%) and G-negative P(8) (25%) followed by G9P(8) (19%), G1P(8) (13%) and G3/G2 P(8), G12P(8), and G-negative P(6) (6% each).

**Conclusion:**

Diarrheal outbreak which occurred in West-Shewa zone of Ethiopia was associated with rotavirus and relatively more affected districts with low vaccination coverage. Routine rotavirus vaccination quality and coverage should be evaluated and the surveillance system needs to be strengthened to detect, prevent and control a similar outbreak.

## Introduction

Rotavirus is a genus *Rotavirus* in the family *Reoviridae* and causes severe diarrhea and vomiting in infants [1]. Rotavirus has a genome consisting of 11 segments of double-stranded RNA [2, 3]. Most segments encode a single polypeptide, allowing the virus to express six structural viral proteins (VP1-VP7) and six nonstructural proteins (NSP1- NSP6) [3]. Rotavirus-infected persons shed high concentrations of rotavirus in the stool. The disease transmits from an infected person to another by fecal-oral route through close person-to-person contact and by fomites. Less commonly, the virus is transmitted by consuming contaminated water or food [4]. Rotavirus is stable and may persist viably in the environment for weeks or even for months if not disinfected [4-6]. Individuals infected by rotavirus disease manifest some watery diarrhea of limited duration to severe diarrhea with vomiting and fever that can result in dehydration with shock, electrolyte imbalance and even death [7]. Following an incubation period of 1-3 days, the illness often begins shortly and vomiting frequently precedes the onset of diarrhea. The gastrointestinal symptoms usually resolved in 3-7 days after the first onset of the illness [8]. Rotavirus is the most common cause of severe, dehydrating gastroenteritis in infants and young children worldwide [9]. An estimated 138 million annual cases and 215,000 deaths in under five children reported in both developing and developed countries, principally in Asia and sub-Saharan Africa [10-12]. Rotavirus infection accounts for 40% of childhood gastroenteritis hospitalizations and 37% of diarrhea-related deaths in children under five years old [13]. Diarrheal disease is a leading killer and causing approximately 16 percent of deaths in children less than five years of age in Ethiopia [14]. Rotavirus is one of the top diarrhea diseases and affect the lives of more than 28,000 Ethiopian children of under five years old every year [15] which makes Ethiopia one of the five developing countries accounted for more than half of all rotavirus deaths: The Democratic Republic of the Congo, Ethiopia, India, Nigeria and Pakistan [16, 17].

Diarrheal diseases caused by rotavirus cannot be clinically differentiated from that caused by other enteric pathogens; specific diagnosis needs testing of fecal specimens with commercially available assays [7]. ELISA, Test device and Latex Agglutination tests are a sensitive diagnostic test for rotavirus detection [18]. However, PCR provided the best overall sensitivity and specificity [19]. This diagnostic testing technique is not usually available for routine patient management. At the hospital level, routine laboratory confirmation is not usually performed as the clinical management mostly relies on appropriate rehydration therapy. Hospital-based rotavirus surveillance in under five children was initiated in 2007 in Addis Ababa to estimate the burden of rotavirus gastroenteritis in children less than five years of age. Studies have shown that rotavirus accounts for 18%-28% of diarrhea hospitalizations among children < 5 years of age in Ethiopia [20-23]. Ethiopia introduced the Rotarix vaccine into its routine immunization program in November 2013 with two doses in 6 and 10 weeks of age [15, 24]. The WHO required 90% and 80% vaccination coverage at the national and district level respectively for all vaccine-preventable diseases including rotavirus [25]. In Ethiopia, the national rotavirus vaccination coverage was 56.0% while it was 50.2% in Oromia region that included West Shewa in 2016 [26]. In pre-vaccine period (2007-2011), the most prevalently detected genotypes in Addis Ababa were G1P(8) (20%), G12P(8) (17%) and G3P(6) (15%) [27]. In the 2^nd^ week of December 2016, West Shewa zone of Oromia regional state reported rotavirus suspected acute diarrhea outbreak. As the number of daily cases increasing the investigation was warranted and conducted to identify the underlying causative agent of the outbreak to support outbreak management and response operations.

## Methods

West Shewa zone is one of the zones in Oromia regional state of Ethiopia to the west of Addis Ababa. Based on the 2007 Census conducted by the Central Statistical Agency of Ethiopia (CSA), this Zone has a total population of 2,058,676, of which 1,028,501 are men, and 1,030,175 are women. The investigation was warranted by the Ethiopian Public Health Institute as part of outbreak response and management. Subsequent permissions obtained from Oromia Regional Health Bureau, West Shewa Health Department, Nono, Danno and Jibat districts health offices to investigate the outbreak. The outbreak case definition for rotavirus was “any person presenting to a health facility with acute diarrhea and/ or vomiting or any person in which clinician suspected rotavirus in Nonno, Danno, Jibat districts after December 16, 2016.” Clinicians were reported cases met the case definition for the duration of the outbreak. Medical records were reviewed and all cases were line listed up on getting verbal permission from medical directors of health facilities. Patients' age, outbreak area setting, contact history, and date of symptoms of onset were used to epidemiologically link gastrointestinal diarrheal cases with laboratory-confirmed rotavirus cases. The descriptive cross-sectional study design was employed, and data analysis was done by time, person and place using Microsoft Excel. The incident rate was calculated by dividing the number of the case to the population (Source: 2007 Census) and expressed per 1,000. The age-specific incident rates were also calculated by dividing the number of cases within a specific age group or sex to respective age group population and multiplying by 1,000. Three years of routine rotavirus vaccination coverage data were received from West Shewa Health Department and compared with suspected rotavirus incidence rate. Incident rate and vaccination coverage were depicted on the map using the Arc Geographic Information System (Arc GIS) 10.4.1 version to illustrate the most affected districts.

Rotavirus infection was determined by using an antigen capture enzyme immunoassay (EIA; ProSpecT^TM^Rotavirus kit, Oxoid Ltd, United Kingdom) on the collected 19 fecal specimens at the national laboratory of the Ethiopian Public Health Institute (EPHI). RT-PCR was used as a gold standard for further confirmation and genotyping. All positive samples were further characterized by molecular methods at the Rotavirus Regional Reference Laboratory (RRRL): South Africa Medical Research Council (SAMRC) Diarrheal Pathogens Research Unit, Department of Virology, SefakoMakgatho Health Sciences University, Pretoria, South Africa. As described before, the VP7 and VP4 genes were amplified by reverse transcription polymerase chain reaction (RT-PCR) using the outer primer sets sBeg/End9 and Con2/Con3 [28, 29]. RT-PCR products for each gene were genotyped using type-specific primers such as G1-G4, G8-G9, and G12 for VP7 gene and primers P(4), P(6), P(8) and P(14) for VP4 gene, respectively. The investigation was conducted to support the response operations or management of the outbreak by identifying the causative agent so as to pinpoint and specify the interventions. Ethiopian public health Institute is mandated by the Council of Ministers to conduct diseases surveillance, epidemiological and laboratory investigations and respond to the outbreaks [30].

## Results

**Figure 1:**
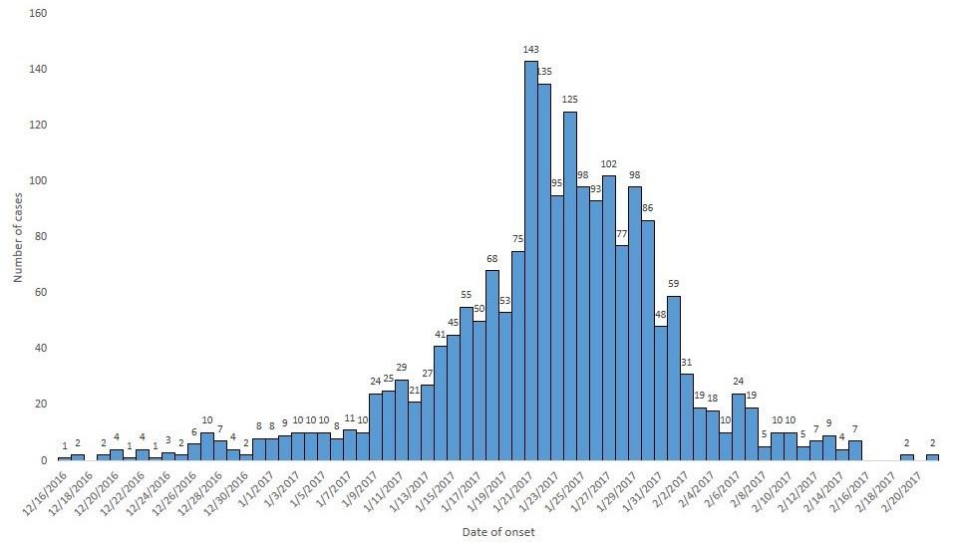
Trend of rotavirus-associated diarrheal cases by date of onset, West Shewa Zone, Oromia region, Ethiopia, 2017

**Figure 2:**
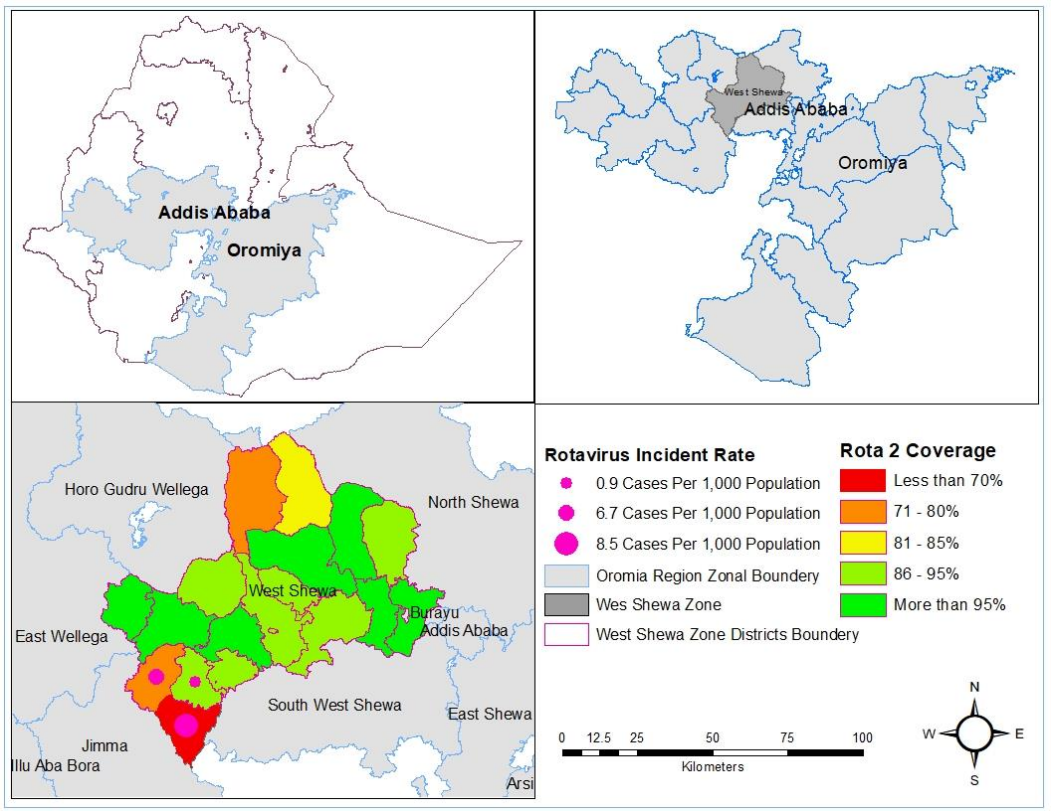
Map of Ethiopian showing rotavirus incidence rate and rotavirus vaccination coverage by districts, 2017

A total of 1,987 diarrheal cases (5.7 per 1,000) and five deaths (case fatality rate 0.25%) were identified and epidemiologically linked to confirmed rotavirus from 16 December 2016 to 21 February 2017. Among the cases, 1121 (56%) were male, and the rest of 866 (44%) cases were female (Table 1). Only 32 (1.6%) of the cases were hospitalized. The majority of the cases, 1946 (98%), were under the age of five years old with nine months median age and interquartile range 14 months (Q_1_ = 6 months and Q_3_ = 20 months). A total of 19 stool specimens were collected during the diarrhea outbreak in the affected districts. All children sampled were with unknown rotavirus vaccination history. Among the collected samples, 14 (74%) were positive for rotavirus by EIA. Male and female contribute 7 (50%) each of the positive cases (Table 1). With regard to genotyping results, majority of the strains found were G12P(6) (25%) and G-negative P(8) (25%) followed by G9P(8) (19%),G1P(8) (13%) and G3/G2 P(8), G12P(8) and G-negative P(6) (6% each), respectively. The crude incidence rate was 5.7 cases per 1,000 populations with some variation among different affected districts. Nonno is the most affected district with the incident rate of 8.5 cases per 1,000 populations. The age group specific incident rate showed that young children less than five years of age were the most affected age groups with the incident rate of 38.2 cases per 1,000 populations of the same age (Table 2). The outbreak started in the 2^nd^ week of December 2016 in Nonno district. It was further spread to Danno and Jibat districts of West Shewa zone and increased to reach high pick on the 3^rd^ week of January 2017 and the number of daily cases started dropping in the same week until the 3^rd^ week of February 2017 (Figure 1). Among total cases, 1224 (62%) were not vaccinated for rotavirus; 693 (35%) were vaccinated while the vaccination statuses of 70 (4%) of the cases were not known (Table 3). Routine rotavirus vaccination coverage was relatively low in Nonno district followed in Danno District over years (Table 4). The 2016/2017 routine 2^nd^ dose vaccination coverage and incidence rate showed in Figure 2.

**Table 1:** Rotavirus cases, deaths and laboratory specimen results by district, West Shewa Zone, Oromia Region, Ethiopia, 2017

District	Cases			Deaths	Laboratory test results		
	Total	Female	Male		Sample	Positive	Positivity Rate
Danno	914	395	519	0	11	7	63.6%
Nonno	983	436	547	0	-	-	-
Jibat	90	35	55	5	8	7	87.5%
**Total**	**1987**	**866**	**1121**	**5**	**19**	**14**	**73.7%**

**Table 2:** Rotavirus cases incident rate per 1,000 populations by age groups and districts, West Shewa Zone, Oromia Region, Ethiopia, 2017

District	0-4 Years	5-14 Years	15-44 Years	45 and above Years	Total
Danno	887 (44.6%)	13 (0.3%)	13 (0.2%)	1 (0.1%)	914 (6.7%)
Nonno	971 (57.7%)	7 (0.2%)	5 (0.1%)	0 (0.0%)	983 (8.5%)
Jibat	88 (6.2%)	2 (0.1%)	0 (0.0%)	0 (0.0%)	90 (0.9%)
Total	1946 (38.2%)	22 (0.2%)	18 (0.1%)	1 (0.0%)	1987 (5.7%)

**Table 3:** Rotavirus vaccination status of the cases, West Shewa Zone, Oromia Region, Ethiopia, 2017

District	Unknown	Unvaccinated	Vaccinated	Total
Danno	4 (0.4%)	755 (82.6%)	155 (16.9%)	914 (100%)
Nonno	19 (1.9%)	452 (46.0%)	512 (52.1%)	983 (100%)
Jibat	47 (52.2	17 (18.9%)	26 (28.9%)	90 (100%)
**Total**	**70 (3.5%)**	**1224 (61.6%)**	**693 (34.9%)**	**1987 (100%)**

**Table 4:** Routine rotavirus vaccination administrative coverage by district, West Shewa Zone, Oromia Region, Ethiopia, 2014-2017

District	2014/2015		2015/2016		2016/2017	
	1st dose %	2nd dose %	1st dose %	2nd dose %	1st dose %	2nd dose %
**Danno**	106	95	101	88	87	72
**Nonno**	97	92	99	81	76	67
**Jibat**	101	96	106	98	106	95
**Total**	**101**	**94**	**101**	**88**	**87**	**76**

## Discussion

This is first reported and investigated rotavirus-associated diarrheal outbreak occurred in three districts of West Shewa Zone of Oromia Region in Ethiopia. The diarrheal outbreak affected the health of many children is associated with rotavirus where 62% of the cases were not vaccinated. In sub-Saharan Africa 45.5% in 2000 and 38.9% in 2013 deaths of diarrheal diseases attributed to rotavirus [31]. Children aged less than five years old were most affected (38 cases per 1,000 populations) by the outbreak with a median age of nine months. Children under five years of age are most vulnerable to the disease [10]. In low-income countries, the median age at the primary rotavirus infection ranges from 6 to 9 months [32, 33]. Similar to our observation, in the Solomon Islands at which the highest attack rate during the outbreak occurred in the < 5 years age groups (32%), which was > 14 times higher than in the ≥ 5 years age groups (2%) [34]. During this outbreak children under five years account for 98% of the total cases which is supported by a study conducted in South Tarawa, Kiribati, that indicates 93.4% of the cases attributed to the rotavirus outbreak and all deaths were under five years old [10]. AS compared with the 2.5% rotavirus CFR estimated by WHO, the CFR we reported here is low [35]. Another study in Ethiopia also reported 2.4% CFR due to rotavirus which is higher as compared to our report [36]. The lower CFR in our investigation might be attributed to the early detection and treatment of cases at health facilities. In our investigation, we observed G12P(6) and G-negative P(8), G9P(8) were circulating dominantly followed by G1P(8), G3/G2 P(8), G12P(8), and G-negative P(6) in the outbreak areas. Whereas, the previous study showed that G3P (6), G1P (8) and G2P (4) are common strains of rotaviruses circulating in Ethiopia [10, 22, 31, 37]. In rural Southern Ethiopia, it was reported that 43.6% of children less than five years of age had diarrhea as a result of rotavirus [38].

G-P combinations such as G1P(8), G2P(4), G3P(8), G4P(8) and G9P(8) contribute about 90% of all human rotavirus infections in which G1P(8) is the most prevalent combination worldwide. Surveillance studies indicate that G1P(8) strain contribute 52.2% of rotavirus strains globally and 17.4% in Africa [39]. Several rotavirus types are circulating in Asia and Africa with greater strain diversity. Among the total rotavirus cases, 52.1%, 28.9% and 16.9% cases were reported with the history of at least one dose of rotavirus vaccination in Nonno, Jibat and Danno districts respectively. The proportion of infected vaccinated children is high in Nonno district which might be attributed to the quality of vaccination. The study conducted in the Republic of Moldova indicates that 25% moderate to severe cases received at least one dose of rotavirus vaccine [40]. The case-control studies conducted in Malawi and Botswana indicate that rotavirus vaccine efficacy for two doses were 64% and 54% respectively which is low compared with developed countries [41, 42]. The administrative vaccination coverage for the last three years varies from district to district. In contrast to the vaccination status of the patients, the vaccination coverage was low in Nonno district. Nonno was the most affected district with a high number of cases and incidence rate. In the district vaccination coverage for the 2^nd^ dose of rotavirus vaccine was 81% in 2015/2016 and 67% in 2016/2017 which was less than the World Health Organization's minimum requirement [25, 43]. Similarly, the vaccination coverage in Danno district was less to prevent the outbreak. However, the rotavirus vaccination coverage was high in Jibat district. In Jibat district effectiveness of the vaccine needs to be further studied and evaluated. Even though rotavirus vaccine efficacy is actually high, the effectiveness of rotavirus vaccine is less in developing countries as compared with developed countries [44]. We also believe that, as the reliability of the administrative vaccination concerns, the true vaccination coverage even might be less than what was reported which might be contributing factors for the outbreak.

## Conclusion

The diarrheal outbreak occurred in three districts of South West Shewa zone of Oromia region in Ethiopia was associated with different rotavirus strains and relatively more affected districts with low vaccination coverage. The existing routine rotavirus vaccination needs to be evaluated and the surveillance system needs to be strengthened to detect, prevent and control a similar future outbreak.

### What is known about this topic

- Rotavirus is the most common cause of severe gastroenteritis and responsible for more than one-third of diarrheal diseases related to hospitalizations and deaths in infants under five years old. Currently, there is a safe and effective vaccine to prevent rotavirus infections.

### What this study adds

- Rotavirus caused diarrheal disease outbreak and mostly affected under five years children in West Shewa Zone of Oromia Region of Ethiopia. The occurrence of the outbreak suggested there is an inadequate rotavirus vaccination coverage in the area.

## Competing interests

All Authors declared no competing interests.
